# Exploring the Impact of Model-Informed Precision Dosing on Procalcitonin Concentrations in Critically Ill Patients: A Secondary Analysis of the DOLPHIN Trial

**DOI:** 10.3390/pharmaceutics16020270

**Published:** 2024-02-14

**Authors:** Sarah Dräger, Tim M. J. Ewoldt, Alan Abdulla, Wim J. R. Rietdijk, Nelianne Verkaik, Christian Ramakers, Evelien de Jong, Michael Osthoff, Birgit C. P. Koch, Henrik Endeman

**Affiliations:** 1 Department of Hospital Pharmacy, Erasmus University Medical Center, 3015 GD Rotterdam, The Netherlands; 2 Rotterdam Clinical Pharmacometrics Group, 3015 GD Rotterdam, The Netherlands; 3 Department of Internal Medicine, University Hospital Basel, 4031 Basel, Switzerland; 4 Department of Intensive Care Medicine, Erasmus University Medical Center, 3015 GD Rotterdam, The Netherlands; h.endeman@erasmusmc.nl; 5 Department of Institutional Affairs, Vrije Universiteit Amsterdam, 1081 HV Amsterdam, The Netherlands; 6 Department of Medical Microbiology and Infectious Diseases, Erasmus Medical Center, 3015 GD Rotterdam, The Netherlands; 7 Department of Clinical Chemistry, Erasmus Medical Center, 3015 GD Rotterdam, The Netherlands; 8 Department of Intensive Care, Rode Kruis Ziekenhuis, 1942 LE Beverwijk, The Netherlands

**Keywords:** model-informed precision dosing, procalcitonin, inflammation, biomarker, antibiotics, critically ill, pharmacodynamic target attainment

## Abstract

Model-informed precision dosing (MIPD) might be used to optimize antibiotic treatment. Procalcitonin (PCT) is a biomarker for severity of infection and response to antibiotic treatment. The aim of this study was to assess the impact of MIPD on the course of PCT and to investigate the association of PCT with pharmacodynamic target (PDT) attainment in critically ill patients. This is a secondary analysis of the DOLPHIN trial, a multicentre, open-label, randomised controlled trial. Patients with a PCT value available at day 1 (T1), day 3 (T3), or day 5 (T5) after randomisation were included. The primary outcome was the absolute difference in PCT concentration at T1, T3, and T5 between the MIPD and the standard dosing group. In total, 662 PCT concentrations from 351 critically ill patients were analysed. There was no statistically significant difference in PCT concentration between the trial arms at T1, T3, or T5. The median PCT concentration was highest in patients who exceeded 10× PDT at T1 [13.15 ng/mL (IQR 5.43–22.75)]. In 28-day non-survivors and in patients that exceeded PDT at T1, PCT decreased significantly between T1 and T3, but plateaued between T3 and T5. PCT concentrations were not significantly different between patients receiving antibiotic treatment with or without MIPD guidance. The potential of PCT to guide antibiotic dosing merits further investigation.

## 1. Introduction

Sepsis and septic shock are potentially life-threatening conditions in critically ill patients in whom timely initiation of antibiotic treatment is important to improve survival [1]. In the intensive care unit (ICU), 40–60% of the patients fail to achieve the currently recommended pharmacodynamic target (PDT) of antibiotics [2,3,4,5]. Due to extensive pathophysiological alterations, they are at risk of both over- and underdosing, increasing the chance of significant adverse events and therapeutic failure [6,7].

Therapeutic drug monitoring (TDM) is optimizing antibiotic dosing using measurements of plasma concentrations and could provide individualized dosing recommendations, especially when combined with model-informed precision dosing (MIPD) [8,9]. MIPD is an approach that uses population pharmacokinetic (PK) models to optimize initial dosing and integrates TDM results using pharmacokinetic software to guide subsequent patient-individualized dosing. Different MIPD software has been developed to facilitate its use in clinical practice [10]. However, individualization of antibiotic dosing might not be limited to TDM or MIPD. Biomarkers might provide additional information about treatment response and toxicity. Procalcitonin (PCT) is a biomarker that indicates the presence of bacterial infection and is a marker for severity of disease and resolution of infection [11,12,13,14,15]. Additionally, some studies have demonstrated its potential utility in guiding antibiotic treatment duration, with subsequent decrease in antibiotic consumption [16,17,18,19,20,21,22].

Pharmacokinetic/pharmacodynamic (PK/PD) relationships of beta-lactam antibiotics and fluoroquinolones are based on the relation between the minimum inhibitory concentration (MIC) of the underlying pathogen and a measure of antibiotic exposure as time above the MIC (T_>MIC_) for beta-lactam antibiotics or area under the concentration–time curve (AUC) for fluoroquinolones [9,23]. Current studies mainly focus on the relationship between PK/PD and the MIC of the pathogen, but rarely take into account the host and its immune response to antibiotic treatment as influencing factor. Consequently, the relationship between PK/PDs of antibiotics, plasma drug exposure and the host’s immune response as reflected by biomarkers such as PCT has rarely been described [9]. However, biomarker-guided antibiotic dosing could be a promising next step towards a patient-individual dosing strategy [9,24].

This study aims to explore the association of MIPD with the course of PCT, and the association of PCT with PDT attainment of antibiotics in critically ill patients. For this purpose, we performed a secondary analysis of a multicentre, randomised controlled trial, the DOLPHIN trial, that investigated the impact of TDM and MIPD on clinical outcome of ICU patients [25,26].

## 2. Material and Methods

### 2.1. Study Design

We performed a secondary analysis on data collected from 388 ICU patients of the DOLPHIN trial, a multicentre, open-label randomised controlled trial [25]. The trial aimed to assess whether early MIPD of beta-lactam antibiotics and ciprofloxacin decreases ICU length of stay (LOS) compared to standard dosing. The trial was conducted between October 2018 and September 2021 in eight ICUs in the Netherlands. The study was conducted in accordance with the Declaration of Helsinki and approved by the Medical Ethics Committee of the Erasmus University Medical Center (Erasmus MC) Rotterdam (registration number MEC-2017-568, 9 March 2018, title: “Dose Individualization on Antibiotics in ICU patients: to TDM or not to TDM and the effects on outcome (DOLPHIN trial)”; EudraCT 2017-004677-14). Informed consent was obtained from all subjects or their legal representatives involved in the study before randomisation. The measurement of PCT was included into the study protocol [26]. Patients were included and randomised within 36 h of the initial administration of the antibiotic (T0). For patients randomised to the MIPD group, the first TDM (T1) was performed within 36 h after randomisation followed by a dosing recommendation within the subsequent 12 h. Subsequently, TDM was performed at day 3 (T3), day 5 (T5), and day 7 (T7) combined with dosage recommendations after T3 and T5. PCT measurements were performed retrospectively, in batches, at the Erasmus MC Rotterdam in patient samples available according to the TDM schedule at T1, T3, T5, and T7. In both trial arms, antibiotic treatment was initiated using standard dosages [25].

### 2.2. Study Populations 

Study population I (*n* = 351): Inclusion criteria for this study population were all patients who participated in the DOLPHIN trial and who had at least one PCT measurement performed at T1, T3, or T5. 

Study population II (*n* = 306): Inclusion criteria for this study population were all patients who participated in the DOLPHIN trial and who had a PCT measurement performed at T1.

Neither T0 nor T7 were included in the study, given the limited significance related to the study setting that investigated the impact of early MIPD and the limited number of measurements available.

### 2.3. Data Collection and Definitions

The demographic data were prospectively collected in the electronic Case Report Form of the original DOLPHIN trial and have been used for this secondary analysis. Clinical characteristics were collected at the time of initiation of antibiotic treatment. The definition of sepsis and septic shock was based on the sepsis III criteria [27]. Sequential Organ Failure Assessment (SOFA) score and laboratory results were assessed at each sample collection. The percentage of PCT decrease (delta PCT) between T1 and T3 and T1 and T5 was calculated as $\frac{T3-T1}{T3}\times 100\;and\;\frac{T5-T1}{T5}\times 100$. Target attainment was defined as achieving PDT of the respective antibiotic after reaching a steady state. The following formula was used to assess PDT for beta-lactam antibiotics:

$$\frac{\frac{Cfree}{Ctotal}\times Cmin\;steady\;state}{Epidemiologic\;cutoff\;value\;(ECOFF)}$$

C_free_: unbound antibiotic concentration; C_total_: total antibiotic concentration; C_min_: minimal concentration, ECOFF: epidemiological cut-off value.

Values < 1 were defined as “below target”, values 1–10 were considered “attained target”, and values > 10 were defined as “above target” corresponding to 100% *f*T_>1×ECOFF,_ 100% *f*T_>1to10×ECOFF,_ and 100% *f*T_>10×ECOFF_. The ECOFFs were used according to the original study [25]. For the analysis of the association between PDT attainment and PCT, patients were allocated to the “above target”/”attained target”/”below target” group at T1 and remained allocated to the corresponding group at T3 and T5. This approach aimed to analyse PCT concentration over time according to the initial PDT. For ciprofloxacin, an AUC over 24 h to the ECOFF (AUC 0–24 h/ECOFF) was used to define PDT. A ratio < 125 h was defined as “below target”, 125–500 h as “attained target”, and >500 h as “above target”. 

### 2.4. Laboratory Methods

EDTA samples were stored at −80 °C in a biobank at the Erasmus MC. After the study termination, PCT was measured in batches in all EDTA samples available at T1, T3, T5, and T7. The measurements were performed using an electrochemiluminescence Brahms PCT immunoassay on a Cobas E801 analyser (Roche Diagnostics, Rotkreuz, Switzerland). 

### 2.5. Outcomes

The primary outcome was the absolute difference in PCT concentration at T1, T3, and T5 between the MIPD and the standard dosing group Secondary outcomes included (i) the relative difference of PCT decrease (delta PCT) between T1 and T3 and between T1 and T5 between the study groups, (ii) the percentage of patients who experienced an ≥80% decrease of PCT concentrations between T1 and T3, and between T1 and T5 or who reached an absolute PCT value < 0.5 ng/mL at T1, T3, or T5 in the MIPD and standard dosing group, respectively, indicating resolution of infection according to the literature [28], (iii) the absolute difference in PCT concentrations at T1, T3, and T5, when only patients with PCT > 0.5 ng/mL were included, (iv) the association between PCT concentrations at T1, T3, and T5 and 28 d mortality presented as 28-day survivors and non-survivors, and (v) the association of PCT concentrations with PDT. 

### 2.6. Statistical Analyses

Continuous data were summarized or as medians with interquartile ranges (IQR). Normality of distribution of PCT at T1, T3, and T5 was tested using the Kolmogorov–Smirnov test due to an appropriately large sample size. As the *p*-value of the Kolmogorov–Smirnov test was <0.001 for T1, T3, and T5, PCT values were log-transformed for further analyses and for the figures. Categorical variables were described using frequencies and percentages. To assess differences between the MIPD and the standard dosing arm, *χ*2 tests were used for categorical data, independent *t*-tests for continuous normally distributed variables, and Mann–Whitney U tests for continuous non-normally distributed variables. To assess differences between two dependent variables (e.g., PCT values at two points in time in one study arm), Wilcoxon signed rank test was used. The primary outcome and the secondary outcome were presented as median with IQR. A two-sided *p*-value < 0.05 was considered statistically significant. All analyses were performed in the R statistical software (version 4.2.1) and Prism Version 9 (GraphPad Software, San Diego, CA, USA). During the analysis phase, we found a downward slope trend in PCT over the study period, though there was no statistically significant difference between the standard dosing and the MIPD study arm. For the analyses between PCT and PDT attainment at T1, and the course of PCT in 28-day survivors and non-survivors, we therefore decided to pool the data from both treatment groups. 

## 3. Results

### 3.1. Patient Characteristics

In total, 662 PCT measurements in 351 patients (study population I) were analysed: 336/662 (50.8%) PCT concentrations in 177 patients were allocated to the standard dosing, and 326/662 (49.2%) PCT concentrations in 174 patients were allocated to the MIPD group, respectively. In this study population, patient characteristics were balanced between both groups (Table 1). 

The median age was 64 years (IQR 55–71) and 132/351 patients (37.6%) were female. There was no significant difference in severity of disease between the patients at admission in regard to APACHE IV score [70 (IQR 51–90) vs. 70 (IQR 51–89), *p* = 0.703] or SOFA score [8 (IQR 5–10) vs. 8 (IQR 5–11), *p* = 0.909]. In total, 200/351 (57.0%) patients presented with sepsis or septic shock. The main focus of infection was the respiratory tract in 234/351 (66.7%) patients and the majority received a beta-lactam antibiotic as empirical treatment (Table 1). There was a slightly, but not statistically significant, shorter median ICU LOS in the standard dosing group compared to the MIPD group (8 days (IQR 3–19) vs. 11 days (IQR 5–20.75), *p* = 0.052), but no significant difference in 28-day mortality (24.9% vs. 25.9%, *p* = 0.902) or 6-month mortality (32.2% vs. 35.6%, *p* = 0.501) (Table 1). 

### 3.2. The Course of PCT

**Figure 1:**
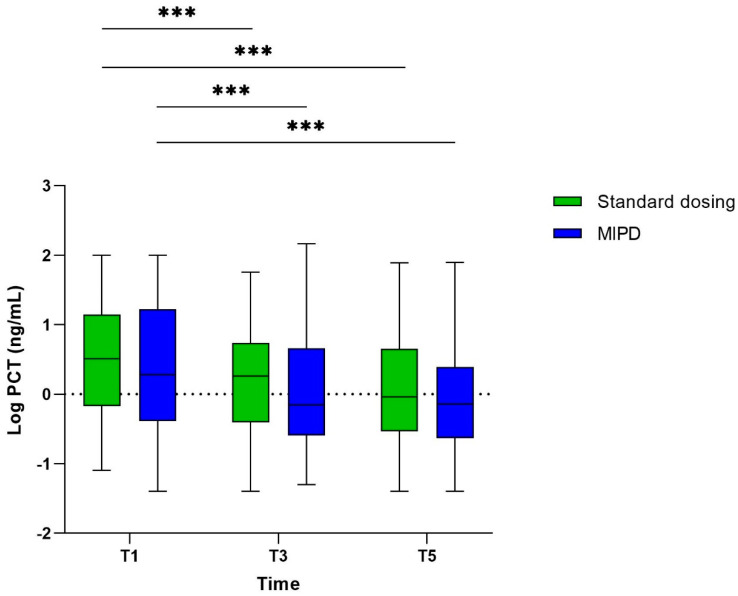
Median procalcitonin concentration at T1, T3, and T5 according to the trial arm. Due to non-normal distributed data, logPCT values are provided. Asterisks indicating statistical significance (<0.0001). PCT: procalcitonin, MIPD: model-informed precision dosing.

The median PCT concentration at T1 was 3.2 ng/mL (IQR 0.7–14.1) in the standard dosing group and 1.9 ng/mL (IQR 0.4–16.6) in the MIPD group. Although the difference in PCT concentrations was not statistically significant between the groups (*p* = 0.153), a tendency towards a higher median PCT concentration in the standard dosing group could be observed (Figure 1). 

In both study arms, PCT decreased significantly over time. Between the study allocations, no significant difference in PCT concentrations could be observed at T3 and T5. There was a slightly greater, but statistically not significant, difference in PCT concentrations between the MIPD and standard dosing group at T3 [PCT standard dosing group 1.83 ng/mL (IQR 0.39–5.30) vs. PCT MIPD group 0.7 ng/mL (IQR 0.26–4.43), *p* = 0.057], which was no longer observed at T5 [PCT standard dosing group 0.91 ng/mL (IQR 0.29–4.44) vs. PCT MIPD group 0.72 ng/mL (IQR 0.24–2.42), *p* = 0.333]. Furthermore, there was no significant difference in the median CRP and white blood cell counts between T1, T3, and T5 in both study arms (Table 1). A PCT value < 0.5 ng/mL at T1 was observed in 75/306 patients (24.5%) at T1. When excluding them from the primary analysis, still no differences in the absolute PCT concentrations between the standard dosing and the MIPD group could be observed, either at T1, T3, or T5. (e.g., PCT T1 standard dosing arm: 6.20 ng/mL (IQR 2.0–18.2) vs. MIPD group 4.44 ng/mL (IQR 1.44–22.93), *p*-value: 0.50) (Supplementary Figure S1).

**Figure 2:**
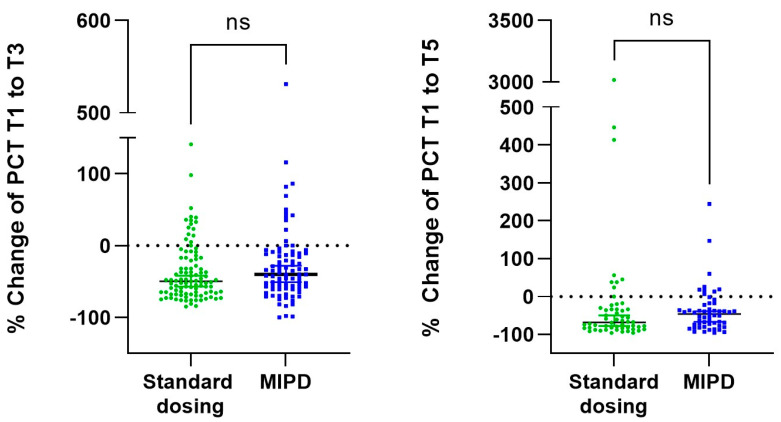
Change of PCT in % in the standard dosing and the MIPD group. ns: not statistically significant. PCT: procalcitonin, MIPD: model-informed precision dosing.

In total, there were 184 and 108 PCT pairs available to assess delta PCT between T1 and T3, and between T1 and T5, respectively. The median PCT decrease was 46% (IQR 13–55) between T1 and T3, and 58% (IQR 31–81) between T1 and T5. There was no difference in PCT decrease (delta PCT in %) between the standard dosing and MIPD group during these periods (Figure 2). Overall, a PCT decrease of ≥80%, which indicates resolution of infection, was observed in 9/184 (4.9%) patients (6 patients in the standard dosing, and 3 patients in the MIPD group) between T1 and T3 and in 29/108 (26.9%) patients (18 patients in the standard dosing and 11 patients in the MIPD group) between T1 and T5. 

### 3.3. Course of PCT in 28-Day Survivors and Non-Survivors (Study Population I)

**Figure 3:**
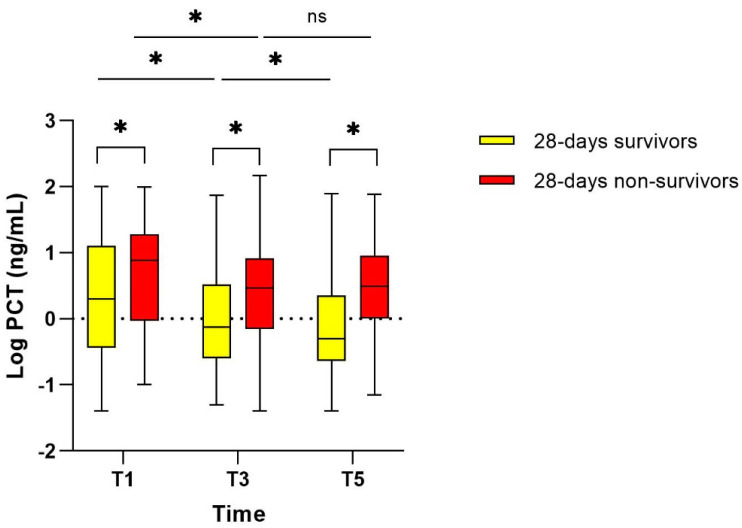
Course of PCT in 28-day survivors and non-survivors. Due to non-normal distributed data, log PCT values are provided. Asterisks indicating statistically significant change. Number of observations (PCT pairs available) 28-day survivors: T1/T3: 140, T1/T5: 81, T3/T5: 89. 28-day non-survivors: T1/T3: 44, T1/T5: 27, T3/T5: 28. ns: not statistically significant. PCT: procalcitonin.

In study population I, there was a statistically significant difference in the median PCT concentration between patients who survived until day 28 and those who died before day 28, at every point in time (Figure 3). In 28-day survivors, the PCT concentration decreased significantly over time between T1 and T3, T1 and T5, and T3 and T5. In contrast, PCT decreased significantly between T1 and T3 (*p* = 0.0001) in 28-day non-survivors, but not between T3 and T5 (*p* = 0.39) (Figure 3). This observation was consistent when analysing the MIPD and standard dosing group separately (Supplementary Figure S2).

### 3.4. Association of PCT with Pharmacodynamic Target Attainment (Study Population II)

**Figure 4:**
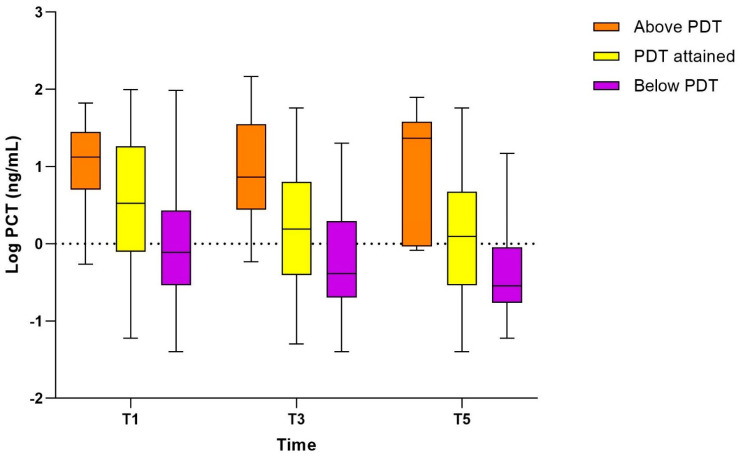
Median procalcitonin concentration at T1, T3, and T5 according to pharmacodynamic target attainment at T1. *n* = 306 patients. Patients were allocated to the PDT group at T1 and were not re-classified at T3 or T5. Due to non-normal distributed data, logPCT values are provided. PCT: procalcitonin; PDT: pharmacodynamic target.

We included 306 patients (study population II) who had a PCT measurement available at T1 into this analysis. One patient out of the initial 307 patients had a PCT measurement available at T1, but did not have any TDM and MIPD performed and was subsequently excluded from this analysis. Baseline characteristics are summarized in Table 2. Of the 306 patients, 178/306 (58.2%) attained PDT, 100/306 (32.7%) were below, and 28/306 (9.2%) exceeded PDT. The median PCT concentration differed significantly between the three PDT groups at T1: the median PCT was 3.35 ng/mL (IQR 0.80–17.52) in patients who attained PDT, 0.76 ng/mL (IQR 0.29–2.41) in patients below, and 13.15 ng/mL (IQR 5.43–22.75) in patients above PDT (*p* < 0.001) (Figure 4 andTable 2). In patients who either achieved or fell below PDT, a significant decrease in PCT over time was observed. However, patients who exceeded PDT at T1 showed a significant difference in PCT between T1 and T3 (*p* = 0.001), but not between T3 and T5 (*p* = 0.578). Similar results were observed when analysing each trial arm separately, although this analysis was limited by the smaller number of observations (Supplementary Figure S3).

Patients who either achieved or exceeded PDT at T1 exhibited higher disease severity, as indicated by higher median APACHE IV and SOFA scores upon admission (Table 2). There was a significant difference in the mortality rate between the PDT groups. Patients that were below PDT had a 28-day mortality rate of 17% (17/100), whereas patients that were on or above PDT demonstrated a 28-day mortality rate of 28.1% (50/178) and 42.9% (12/28), respectively (*p* = 0.01).

## 4. Discussion

We performed a secondary analysis on data from the DOLPHIN trial, in which we observed a significant decrease in PCT over the study period in the MIPD and the standard dosing study arm. In addition, patients exceeding the predefined PDT at T1 were found to have higher PCT concentrations in the blood, had poorer clinical outcome, and showed persistant elevated PCT values after an initial response to antibiotic treatment.

We did not find a difference in absolute PCT concentrations or the decrease of PCT over time in critically ill patients who received antibiotic treatment with or without MIPD and TDM guidance. Although not statistically significant, we observed a lower PCT concentration two to three days after the initiation of the antibiotic treatment in the MIPD group, pointing towards a potential impact of MIPD on a faster resolution of infection. However, the lack of any significant impact of MIPD on the course of PCT is in line with the results of the main study, in which no difference between the study arms was observed with regard to ICU LOS as the primary outcome parameter and mortality [25]. The limitation of the DOLPHIN trial, i.e., the heterogeneity of patients included and the trial design, are still relevant in this secondary analysis and potentially masking a greater effect of MIPD on the PCT decrease, i.e., the resolution of infection [29,30,31].

Although the association between the PK/PD of antibiotics and the host response has been discussed in the literature [9,13,23,24,32], clinical studies investigating this relationship are scarce, with no large prospective observational or randomised controlled trial data available to date. Aldaz et al. conducted a retrospective, observational, controlled cohort study with 96 patients in total, in which the decrease of PCT ≥ 80% until the end of treatment was assessed to describe the effectiveness of TDM-guided meropenem treatment [33]. A significantly higher proportion of patients in the TDM-guided group (71%) showed a PCT decrease of ≥80% by the end of the treatment compared to those who received meropenem treatment without TDM guidance (53%; *p* = 0.02). In contrast, our study did not reveal any difference in PCT decrease between the study arms. Moreover, a decrease of PCT ≥ 80% until T3 could only be observed in 9%, and in 25% of the patients until T5, being substantially lower compared to the results of Aldaz et al. [33]. However, comparability of the two studies is limited due to the different time points used to assess the PCT decline, the different antibiotics investigated, and the different study designs. In our study, we observed that approximately a quarter of the patients had a PCT < 0.5 ng/mL at T1. Although the use of PCT to guide initiation of antibiotic treatment is still debated [28], and its use in guiding the moment to stop antibiotic treatment is more accepted [15,34], PCT values < 0.5 ng/mL may be associated with a lower probability of bacterial infection in sepsis patients [28]. Overall, this group of patients with PCT values < 0.5 ng/mL might have had less benefit from antibiotic treatment and subsequently from TDM and MIPD.

We found that patients who had a high initial PCT value were at risk of exceeding the 10× PDT and of experiencing potential drug toxicity. Additionally, these patients represented the sickest subgroup of patients, reflected in a high APACHE Score, low eGFR, and high PCT values. Subsequently, they showed a significantly higher 28-day and 6-month mortality rate. This may underline the role of PCT as marker of severity of disease and to predict mortality [35]. Severity of disease is characterized by multi-organ failure due to a hyper-inflammatory status reflected in, e.g., high PCT concentrations, and at the same time decreased hepatic and renal function leading to a decrease of antibiotic clearance [36]. Therefore, it is a risk factor for excessive plasma concentrations of beta-lactam antibiotics, which, in turn, has been shown to be present with higher mortality rates [5,37,38]. However, previous findings were mainly based on clinical scores and altered renal function, but not on biomarker concentrations. Our results underscore the value of PCT as marker of severity of disease and show that higher PCT values are more frequently present in patients exceeding PDT. Additionally, although beta-lactams are known to have a wide therapeutic window, it is unclear if they may cause more toxic side effects than actually presumed [39,40].

It is noteworthy that PCT values plateaued between T3 and T5 in two distinct patient groups, the 28-day non-survivors and those exceeding PDT at T1. Antibiotic treatment seemed to have less impact on the course of PCT in the latter patients in the later state of the infection compared to patients who attained PDT or remained below PDT. The elevated PCT concentrations in these patients may not be, or only partially be, associated with a bacterial infection and be responsive to antibiotic treatment. Hence, it remains unclear if the optimization of antibiotic treatment may result in an improvement of clinical outcome or if it represents a hyper-inflammatory status that requires another treatment approach, e.g., the use of anti-inflammatory drugs which might have a greater impact on a favourable course of the disease. Conversely, patients with lower PCT values might benefit more from TDM and MIPD guided treatment, especially as they frequently did not achieve PDT. Proteomic and metabolomic studies to detect sepsis phenotypes might improve the understanding of the origin of inflammation and the classification of sepsis patients to different subclasses to identify those who may benefit the most from MIPD [41,42]. Furthermore, studies investigating the longitudinal trends of PCT during the course of infection and treatment are required to better understand the dynamics and the role of persisting high PCT plasma concentrations beyond the first phase of treatment.

To better understand the impact of MIPD on infection resolution and the PCT trajectory, prospective studies incorporating PCT measurements in their design are required. Given its more widespread availability compared to TDM and MIPD and its availability as point-of-care test, PCT could play an important role for future TDM trials and dosing strategies, e.g., helping to identify patients being at risk to exceed PDT combined with its role as marker for bacterial infections and severity of inflammation and disease. Furthermore, more research is needed to describe the relationship between inflammation biomarkers, i.e., the host’s immune response, PK/PD parameters and TDM, more precisely. Gatti et al. has shown that inflammation reflected in high plasma CRP and PCT concentrations may down-regulate the metabolism of voriconazole in patients with COVID-19-associated aspergillosis, resulting in potentially toxic voriconazole concentrations [43]. In antibiotics, exposure–response relationships using modelling approaches have been described in pharmacodynamics of vancomycin and CRP in adults [44], of teicoplanin and CRP in neonates [45] or in animal studies [23]. Aulin et al. described the pharmacodynamics of PCT in patients with sepsis who had a daily PCT measurement performed and showed a significant variation of its dynamics as response to antibiotic treatment [24]. We present first data from a large clinical trial providing more insights of the association between PCT and antibiotic PDT attainment underlining the need for future prospective studies. 

This study has several limitations. First, approximately 10% of the patients of the DOLPHIN trial could not be included in this secondary analysis due to the lack of samples available to determine PCT. This may have introduced some selection bias. PCT was not available in all patients at all points in time. Therefore, longitudinal, paired analyses were limited to the patients in whom at least two PCT measurements were available. Due to the study design, PCT was rarely available at T0, the initiation of antibiotic treatment, and therefore was not included into the analysis. We did not include microbiological data to support the role of PCT as a marker of bacterial infection due to its inconsistent collection and limited availability. Furthermore, data regarding concurrent treatments with drugs that could potentially influence the course of PCT were not available. Finally, we used a similar PCT threshold throughout the study population, although it may vary in patients with chronic renal dysfunction or congestive heart failure [18].

## 5. Conclusions

MIPD was not shown to have a significant impact on the course of PCT over time in critically ill patients. Patients with higher PCT values were more likely to exceed PDT and had a higher mortality rate. More severely ill patients demonstrated persistant elevated PCT concentrations after initial response to antibiotic treatment. The potential of PCT as biomarker for the identification of patients who are at risk to exceed PDT, and the use of PCT to guide antibiotic dosing, should be focus of future prospective trials. 

## Figures and Tables

**Table 1:** Baseline characteristics of the study population I.

	Standard Dosing (*n* = 177)	MIPD (*n* = 174)	Total (*n* = 351)	*p*-Value
Age, median (IQR)	64 (54–70)	65 (56–72)	64 (55–71)	0.301
Female sex, *n* (%)	66 (37.3)	66 (37.9)	132 (37.6)	0.913
BMI, median (IQR), kg/m^2^	25.9 (23.0–29.4)	26.3 (23.4–31.1)	26.1 (23.1–30.6)	0.292
CCI, median (IQR)	3 (2–5)	3 (2–4)	3 (2–5)	0.222
APACHE IV Score, median (IQR)	70 (51–90)	70 (51–89)	70 (51–89)	0.703
SOFA Score T1, median (IQR)	7 (4–9)	7 (4–10)	7 (4–10)	0.363
SOFA Score T3, median (IQR)	4 (2–8)	5 (2–8)	5 (2–8)	0.425
SOFA Score T5, median (IQR)	1.5 (0–6)	3 (0–6)	2 (0–6)	0.057
Sepsis, *n* (%)				0.333
No	77 (44)	84 (48)	161 (46)	
Sepsis	56 (32)	58 (33)	114 (33)	
Septic shock	44 (25)	32 (18)	76 (22)	
Antibiotic class, *n* (%)				0.901
Beta-lactam	135 (76)	131 (75)	266 (76)	
Fluoroquinolone	42 (24)	43 (25)	85 (24)	
Main focus of infection, *n* (%)				0.921
Pulmonary	117 (66)	117 (67)	234 (67)	
Intra-abdominal	27 (15)	29 (17)	56 (16)	
Skin and soft tissue	6 (3)	3 (2)	9 (3)	
Central nervous system	5 (3)	4 (2)	9 (3)	
Urinary tract	3 (2)	6 (3)	9 (3)	
Bacteraemia	6 (3)	2 (1)	8 (2)	
Catheter-related infection	2 (1)	2 (1)	4 (1)	
Ear, nose, throat	1 (1)	2 (1)	3 (1)	
Endocarditis	1 (1)	1 (1)	2 (1)	
Unknown focus	6 (3)	5 (3)	11 (3)	
Other	3 (2)	3 (2)	6 (2)	
Laboratory values, median (IQR)				
PCT T1, ng/mL	3.22 (0.71–14.0) *	1.92 (0.41–16.2) **	2.35 (0.54–14.25)	0.153
PCT T3, ng/mL	1.83 (0.39–5.30) *	0.7 (0.26–4.43) **	1.15 (0.34–4.96)	0.057
PCT T5, ng/mL	0.91 (0.29–4.44) *	0.72 (0.24–2.42) **	0.89 (0.25–3.35)	0.333
WBC T1, ×10^9^/L	13.0 (9.2–18.0)	13.6 (8.8–17.4)	13.2 (8.9–17.7)	0.978
WBC T3, ×10^9^/L	12.6 (8.7–16.0)	11.7 (8.7–18.0)	12.2 (8.7–17.1)	0.918
WBC T5, ×10^9^/L	12.8 (9.8–16.6)	13.5 (9.8–18.7)	13.1 (9.7–18.3)	0.439
CRP T1, mg/L	216 (123–329)	197 (104–304)	213 (110–321)	0.176
CRP T3, mg/L	128 (71–223)	122.5 (67–191)	123 (68–200)	0.536
CRP T5, mg/L	84 (42–180)	80 (43–160)	82 (42–169)	0.801
Creatinine T1, µmol/L	94 (63–146)	89 (58–163)	91 (60–153)	0.602
Creatinine T3, µmol/L	85 (59–128)	75 (54–140)	80 (55–135)	0.742
Creatinine T5, µmol/L	84 (55–119)	70 (50–122)	77 (53–120)	0.424
Outcome				
ICU LOS, median (IQR)	8 (3–19)	11 (5–20.75)	10 (4–20)	0.052
Hospital LOS, median (IQR)	21 (10–36.25)	26 (14–43.75)	23 (12.00, 40.75)	0.035
Mortality 28 days, *n* (%)	44 (24.9)	45 (25.9)	89 (25.4)	0.902
Mortality 6 months, *n* (%)	57 (32.2)	62 (35.6)	119 (33.9)	0.501

Patients had a least one PCT measurement available at T1, T3, or T5. MIPD: Model-informed precision dosing; IQR: interquartile range; BMI: body mass index; CCI: Charlson Comorbidity Score; APACHE IV: Acute Physiology and Chronic Health Evaluation version 4; SOFA: sequential organ failure assessment; PCT: procalcitonin, CRP: C-reactive protein; WBC: white blood cell count; ICU LOS: intensive care unit length of stay. * Number of PCT values available: T1 = 161, T3 = 114, T5 = 61. ** Number of PCT values available: T1 = 146, T3 = 108, T5 = 7.

**Table 2:** Baseline characteristics of the study population II (*n* = 306 patients) with a PCT available at T1 allocated to the according target attainment group.

	Below Target (*n* = 100)	Attained Target (*n* = 178)	Above Target (*n* = 28)	Total (*n* = 306)	*p* Value
Age, median (IQR)	61 (49–68)	65 (57–70)	68 (61–73)	64 (55–71)	0.003
Female sex, *n* (%)	33 (33.0)	69 (38.8)	15 (53.6)	117 (38.2)	0.141
BMI, median (IQR), kg/m^2^	25.7 (23.0–29.5)	26.5 (23.2–30.9)	24.0 (20.2–26.2)	26.2 (22.9–30.6)	0.063
CCI, median (IQR)	2 (1–4)	3 (2–4)	4 (3–5)	3 (2–4)	<0.001
APACHE IV Score, median (IQR)	64 (48–85)	73 (56–89)	78 (60–95)	70 (51–89)	0.046
SOFA Score T0, median (IQR)	6 (4–8)	8 (5–11)	9 (8–11)	8 (5–10)	<0.001
SOFA Score T1, median (IQR)	5 (4–8)	8 (4–11)	7 (5–9)	7 (4–10)	<0.001
SOFA Score T3, median (IQR)	4 (2–6)	6 (3–11)	6 (4–10)	5 (3–9)	<0.001
SOFA Score T5, median (IQR)	3 (2–5)	6 (3–10)	6 (3–9)	5 (3–8)	<0.001
Sepsis, *n* (%)					<0.001
No	64 (64)	69 (39)	12 (43)	145 (47)	
Sepsis	29 (29)	63 (35)	9 (32)	101 (33)	
Septic shock	7 (7)	46 (26)	7 (25)	60 (20)	
Antibiotic class, *n* (%)					<0.001
Beta-lactam	53 (53)	147 (83)	28 (100)	228 (75)	
Fluoroquinolone	47 (47)	31 (17)	0 (0)	78 (26)	
Main focus of infection, *n* (%)					<0.001
Pulmonary	82 (82)	113 (64)	11 (39)	206 (67)	
Intra-abdominal	4 (4)	33 (19)	10 (36)	47 (15)	
Skin and soft tissue	3 (3)	5 (3)	1 (4)	9 (3)	
Central nervous system	2 (2)	6 (3)	0 (0)	8 (3)	
Urinary tract	1 (1)	6 (3)	1 (4)	8 (3)	
Bacteraemia	0 (0)	4 (2)	2 (7)	6 (2)	
Catheter-related infection	0 (0)	4 (2)	0 (0)	4 (1)	
Ear, nose, throat	1 (1)	1 (1)	1 (4)	3 (1)	
Endocarditis	1 (1)	1 (1)	0 (0)	2 (1)	
Other	3 (3)	1 (1)	2 (7)	6 (2)	
Unknown focus	3 (3)	4 (2)	0 (0)	7 (2)	
Laboratory values, median (IQR)					
PCT T1, ng/mL	0.76 (0.29–2.41) *	3.35 (0.80–17.52) **	13.15 (5.43–22.75) ***	2.34 (0.53–14.28)	<0.001
PCT T3, ng/mL	0.42 (0.22–1.80) *	1.46 (0.46–5.92) **	3.96 (2.82–10.82) ***	1.07 (0.33–4.18)	<0.001
PCT T5, ng/mL	0.27 (0.17–0.84) *	1.04 (0.28–3.61) **	1.07 (0.89–1.70) ***	0.78 (0.23–2.36)	<0.001
WBC T1, ×10^9^/L	13.6 (9.3–17.8)	12.85 (8.8–17.3)	16.1 (13.6–22.0)	13.6 (9.2–17.9)	0.02
WBC T3, ×10^9^/L	12.0 (8.8–15.4)	12.1 (8.5–17.6)	16.6 (12.3–20.3)	12.4 (8.7–17.2)	0.039
WBC T5, ×10^9^/L	13 (10.3–16.3)	13.7 (9.9–18.9)	16.7 (12.5–24.7)	13.4 (10.0–18.7)	0.169
CRP T1, mg/L	188 (94–288)	214 (107–332)	245 (195–307)	213 (110–322)	0.117
CRP T3, mg/L	112 (58–204)	129 (72–198)	114 (89–177)	121 (67–193)	0.658
CRP T5, mg/L	70 (39–162)	82 (42–175)	78 (68–119)	76 (42–165)	0.875
Creatinine T1, µmol/L	64 (52–91)	105 (68–155)	173 (97–233)	90 (60–149)	<0.001
Creatinine T3, µmol/L	60 (48–82)	89 (59–135)	144 (87–236)	78 (54–128)	<0.001
Creatinine T5, µmol/L	59 (44–81)	82 (56–120)	191 (142–253)	76 (52–118)	<0.001
Outcome					
ICU LOS, median (IQR)	10 (4–19)	10 (4–21)	4 (2.75–9)	9.5 (3.25–19)	0.006
ICU LOS, median (IQR) 28 d survivors	11 (4–21.5)	10 (3–21.5)	4 (2–6.25) ^$^	10 (3–20.5)	0.028
Hospital LOS, median (IQR)	25 (12.5–41.0)	24 (12.25–43.0)	14.5 (8.5–22.25) ^$^	23 (12.0–41.0)	0.027
Hospital LOS, median (IQR) 28 d survivors	28 (14.25–44.5)	29 (15–49)	18 (11.5–38)	28.5 (14.25–47.75)	0.336
Mortality 28 d, *n* (%)	17 (17)	50 (28.1)	12 (42.9)	79 (25.8)	0.010
Mortality 6 months, *n* (%)	21 (21)	71 (39.9)	14 (50.0)	106 (34.6)	<0.001

BMI: body mass index, APACHE IV: Acute Physiology and Chronic Health Evaluation version 4; SOFA: sequential organ failure assessment; ICU LOS: intensive care unit length of stay; CNS: central nervous system; CRP C-reactive protein; WBC white blood cell count. * Number of PCT values available: T1 = 100, T3 = 61, T5 = 36. ** Number of PCT values available: T1 = 178, T3 = 110, T5 = 66. *** Number of PCT values available: T1 = 28, T3 = 12, T5 = 5. ^$^ Number of observations = 16.

## Data Availability

The data that support the findings of this study are available from the corresponding author upon reasonable request.

## Supplementary Materials

The following supporting information can be downloaded at: https://www.mdpi.com/article/10.3390/pharmaceutics16020270/s1: Figure S1. Median procalcitonin concentration at T1, T3, and T5 in patients with a PCT > 0.5 ng/mL at T1 according to the trial arm. Due to non-normal distributed data, logPCT values are provided. Asterisks indicating statistical significance (<0.0001). ns: not statistically significant. PCT: procalcitonin, MIPD: model-informed precision dosing. Figure S2. Course of procalcitonin in 28-day survivors and non-survivors according to the trial arm. Due to non-normal distributed data, log PCT values are provided. PCT: procalcitonin, MIPD: model-informed precision dosing. Figure S3. Course of procalcitonin according to the pharmacodynamic target attained at T1 and according to the trial arm. Due to the limited number of observations, we refrained from further statistical tests. PCT: procalcitonin, MIPD: model-informed precision dosing, PDT: pharmacodynamic target.

## Abbreviations

APACHE IV	Acute physiology and chronic health evaluation version 4
BMI	Body mass index
CRP	C-reactive protein
GFR	Glomerular filtration rate
ICU	Intensive Care Unit
LOS	Length of stay
MIC	Minimal inhibitory concentration
MIPD	Model informed precision dosing
PCT	Procalcitonin
PK/PD	pharmacokinetic/pharmacodynamic
PDT	pharmacodynamic target
RCT	Randomised controlled trial
SOFA	Sequential organ failure assessment
TDM	Therapeutic drug monitoring
