# Supplementary material for: Exploring the Impact of Model-Informed Precision Dosing on Procalcitonin Concentrations in Critically Ill Patients: A Secondary Analysis of the DOLPHIN Trial
Source: Pharmaceutics. 2024 Feb 14;16(2):270. doi: 10.3390/pharmaceutics16020270 (PMC10891837; doi:10.3390/pharmaceutics16020270)

Supplement

Figures

Figure S1. Median procalcitonin concentration at T1, T3 and T5 in patients with a PCT>0.5 ng/mL at T1 according to the trial arm. Due to non-normal distributed data, logPCT values are provided. Asterisks indicating statistical significance (<0.0001). ns: not statistically significant. PCT: procalcitonin, MIPD: model-informed precision dosing.

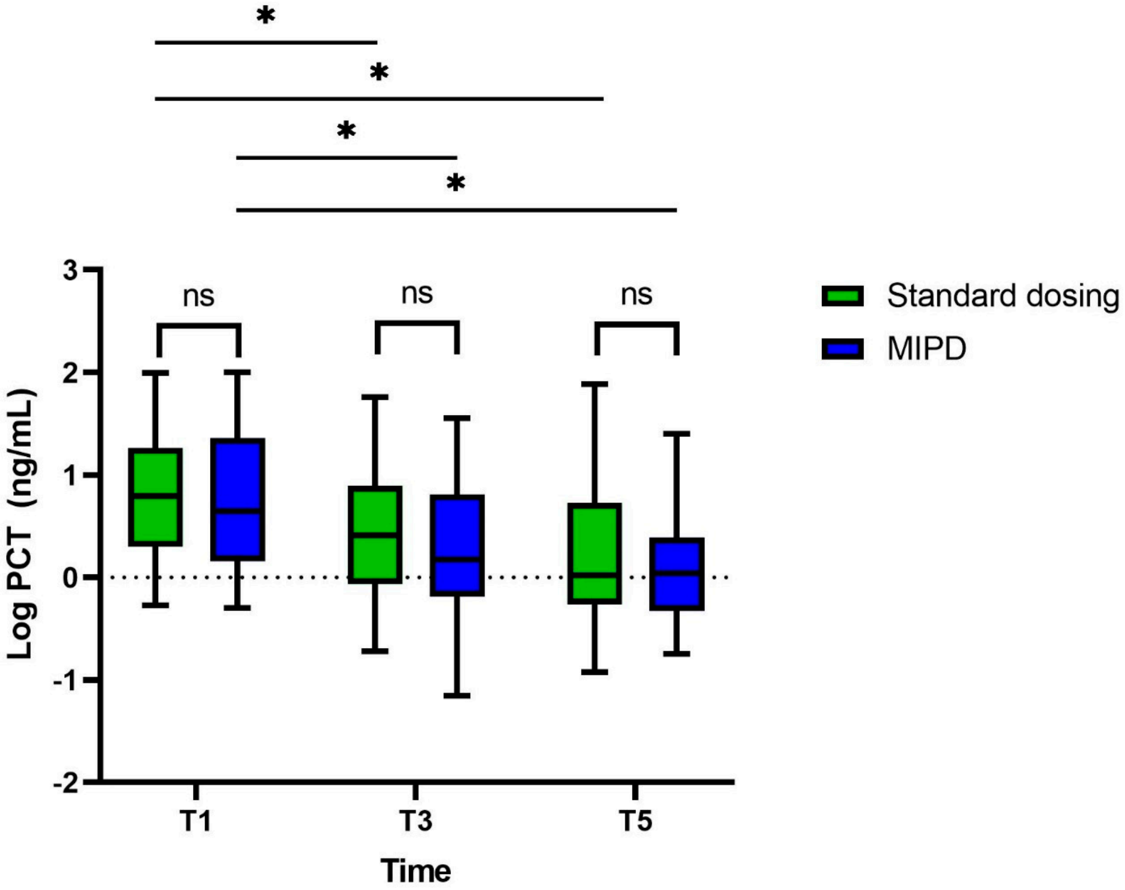

Figure S2. Course of procalcitonin in 28-days survivors and non-survivors according to the trial arm. Due to non-normal distributed data, log PCT values are provided. PCT: procalcitonin, MIPD: model-informed precision dosing

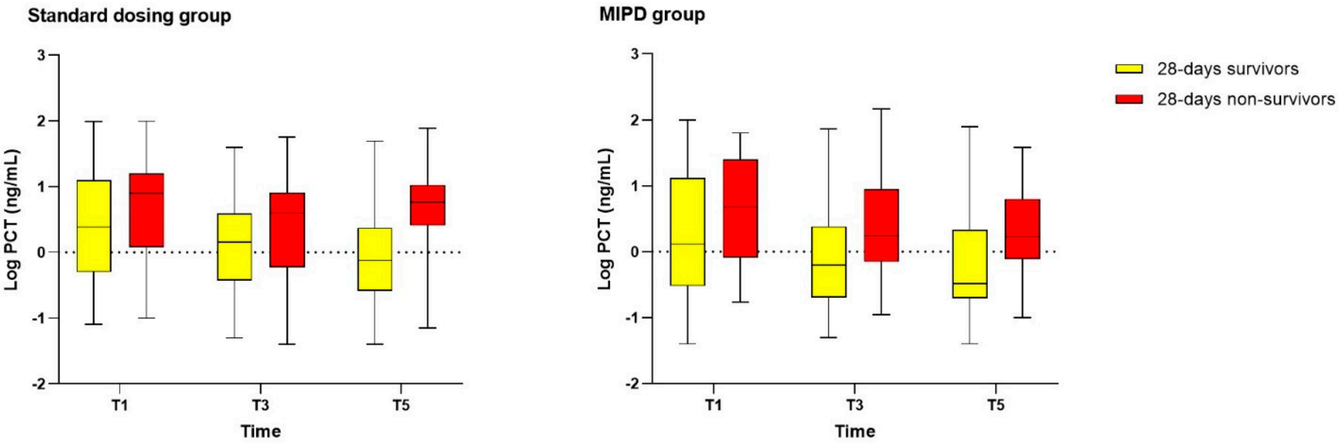

Figure S3. Course of procalcitonin according to the pharmacodynamic target attained at T1 and according to the trial arm. Due to the limited number of observations, we refrained from further statistical tests. PCT: procalcitonin, MIPD: model-informed precision dosing, PDT: pharmacodynamic target

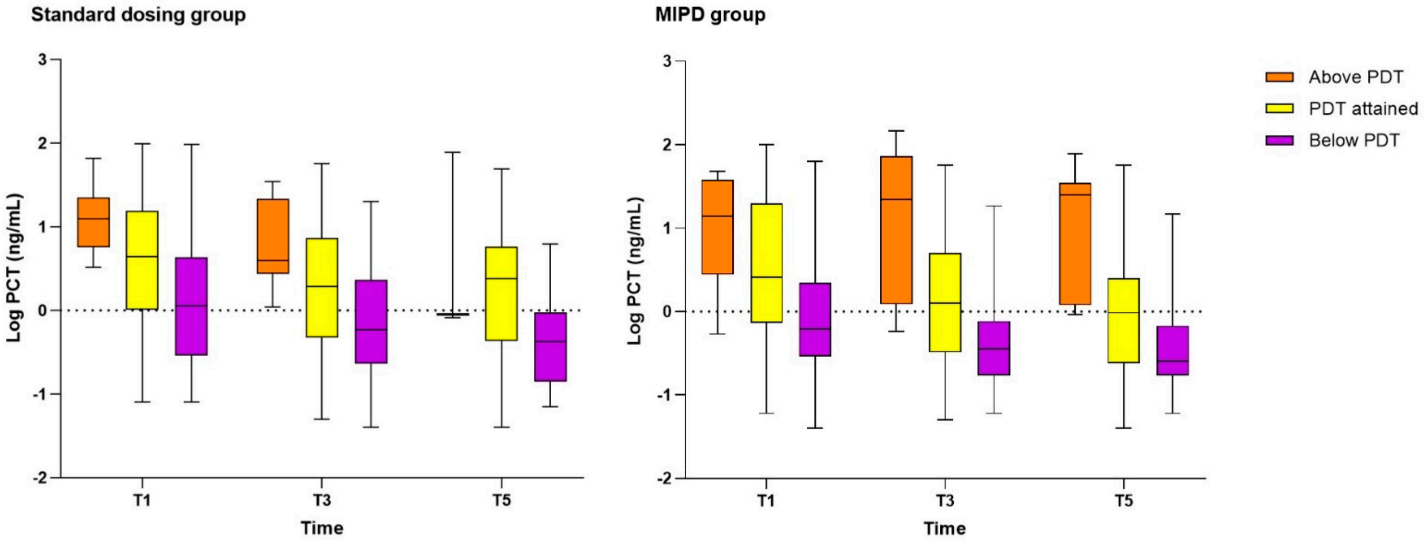

Supplement: Supplementary file 1 [file pharmaceutics-16-00270-s001.zip › pharmaceutics-2731081-supplementary.pdf]
